# Metabolic Profile of *Histomonas meleagridis* in Dwyer’s Media with and Without Rice Starch

**DOI:** 10.3390/metabo14120650

**Published:** 2024-11-22

**Authors:** Sawsan Ammar, Courtney J. Christopher, Nicole Szafranski, Rebekah Jones, Sree Rajeev, Hector F. Castro, Shawn R. Campagna, Richard Gerhold

**Affiliations:** 1 Department of Biomedical and Diagnostic Sciences, College of Veterinary Medicine, University of Tennessee, 2407 River Drive, Knoxville, TN 37996, USA; sawsanibrahimammar@gmail.com (S.A.); nszafran@vols.utk.edu (N.S.); rdjones@utk.edu (R.J.); srajeev@utk.edu (S.R.); 2 Collage of Veterinary Medicine, The University of Sadat City, Sadat City, Menofia 32511, Egypt; 3 Department of Chemistry, Biological Small Molecules Mass Spectrometry Core, University of Tennessee, Knoxville, TN 37996, USA; cleathe3@vols.utk.edu (C.J.C.); hcastrog@utk.edu (H.F.C.); campagna@utk.edu (S.R.C.)

**Keywords:** *Histomonas meleagridis*, metabolomics, mass spectrometry, protozoa propagation, riboflavin

## Abstract

Background and objectives: *Histomonas meleagridis*, the causative agent of histomonosis (i.e., blackhead disease), threatens the poultry industry with serious economic losses due to its high mortality and morbidity in turkey and chicken flocks. In vitro studies are complicated by the inability to culture the parasite axenically. *Histomonas meleagridis* has been propagated in Dwyer’s media, which contains a starch source and serum, for over 50 years. The presence of insoluble starch component in Dwyer’s media represents an obstacle for the commercialization of such media, and the role of starch in media is poorly understood. Methods: To investigate the intracellular metabolomic differences in *H. meleagridis* and undefined bacteria grown in Dwyer’s media with rice starch (SD) and without rice starch (NR), we conducted a global metabolomics analysis using ultra-high-performance liquid chromatography–high-resolution mass spectrometry. Results: SD significantly supported the growth of *H. meleagridis* compared to NR. There was no significant difference in bacterial growth between SD and NR media at various timepoints. From the intracellular metabolic analysis of samples collected from the SD and NR media, a total of 170 known metabolites were identified. *H. meleagridis* appears to be the major contributor to global metabolic differences. Conclusions: We found that riboflavin had the highest variable importance in the projection score, and metabolites involved in riboflavin biosynthesis significantly contributed to the differences between SD and NR in the media immediately after the inoculation of *H. meleagridis* and undefined bacteria, warranting further investigations into the role of riboflavin biosynthesis in *H. meleagridis* growth.

## 1. Introduction

*Histomonas meleagridis* is an important protozoan parasite in birds. The parasite primarily infects gallinaceous birds and causes devastating impacts on turkeys with mortalities reaching up to 100% [1,2]. In chickens, the disease is less severe and may present with a mild decrease in egg production in laying hens, but it is an emerging concern in replacement pullets and broiler breeders [3]. Historically, histomonosis was well controlled with the available treatments, which led to a lack of development of research on *H. meleagridis* except for the basic traditional science on it [4]. Recently, due to the ban of available treatments for the parasite in the European Union and USA, a re-emergence of histomonosis outbreaks has been reported [4]. As a result of this re-emergence, gaps in knowledge and research on *H. meleagridis* were identified, and this led to opportunities for molecular and omics-based research on *H. meleagridis* [5,6]. Importantly, the use of new research strategies to better understand the organism and its growth requirements is warranted.

Dwyer’s media has been used for the last few decades to grow the parasite [7]. Although the original media described by Dwyer contains chick embryo extract, this component was omitted later, leaving the Dwyer’s media with only rice starch, serum, and M199 as the main components [8]. Since then, several manipulations were attempted to increase parasite yield and to elucidate *H. meleagridis* growth requirements [9,10,11]. Starch source and serum are essential components in Dwyer’s media, and *H. meleagridis* is unable to grow in media lacking either component [10,11]. There are ample proposed theories on the role of the rice starch in *H. meleagridis* media. Van der Heijden et al. described how *H. meleagridis* is only able to digest rice starch particles less than five microns in size. They proposed that the bigger particles are probably digested by bacteria to provide *H. meleagridis* with further nutrients [11]. The role played by bacteria is not fully defined. The bacteria are introduced in the cultures through the inoculum of cecal contents, and trials growing axenic *H. meleagridis* cultures have been unsuccessful [12,13].

Understanding the role of rice starch in the media may assist with the ability to modify Dwyer’s media and elucidate the metabolic pathway of the parasite. Also, understanding the small-molecule interactions between the bacteria and histomonads is crucial to identifying the role the bacteria play in *H. meleagridis* growth. Furthermore, understanding the essential nutrient requirements may aid in developing axenic *H. meleagridis* cultures by providing the culture with nutrients generated by the bacteria, which is currently not possible. Having axenic *H. meleagridis* cultures will open doors to various applications and research on the organism and will allow for deeper studies on metabolic pathways that may allow for future drug research and discovery. Using a global metabolomics approach, our goal was to identify essential components required for *H. meleagridis* growth, which would allow us to replace the rice starch with a less complex and more soluble component. We hypothesize that there is a difference in the growth of *H. meleagridis* in Dwyer’s media with (SD) and without rice (NR), which will lead to differences in the metabolic profiles of cells grown in the SD media compared to the NR media. Therefore, this study aimed to identify the key metabolites driving this difference and manipulating them in future experiments to eventually replace the rice starch with a soluble component.

## 2. Materials and Methods

### 2.1. Histomonas meleagridis Strain Preparation

The *Histomonas meleagridis* UGA strain, isolated from a domestic turkey in GA, USA, was used in this study. The isolate was propagated in Dwyer’s media containing M199, sodium bicarbonate, rice starch, and inactivated horse serum at 40 °C in a laboratory and passed multiple times. The isolates were cryopreserved in liquid nitrogen and successfully resuscitated for various experiments.

The strain was incubated in twenty-one T25 tissue culture flasks of Dwyer’s media (SD) at 40–42 °C until logarithmic growth (after 48–72 h). The contents of the T25 flasks were transferred with a plastic pipette to a 500 mL tube. Histomonads were counted in the 500 mL tube using a hemocytometer. The tube was centrifuged at 200× *g* for two minutes, and the supernatant was discarded. The pellet was resuspended in 50 mL of warm Dwyer’s media with no rice added (NR) and centrifuged, and then, the supernatant was discarded. This washing step was repeated once more, followed by a final resuspension in 10 mL of warm NR media. A total of 1 mL of the final suspension (count adjusted to 1.09 × 10^6^ cells/mL) was added to each of the 10 T75 flasks containing 100 mL of either SD (5 flasks) or NR media (5 flasks). The flasks were then immediately sampled (zero sample) and incubated at 40–42 °C for the rest of the subsequent samplings. Dwyer’s media was prepared in-house under non-sterile conditions.

### 2.2. Sample Collection and Preparation

Three T75 flasks containing media (SD n = 3, NR n = 3) were sampled from each treatment before inoculating them with *H. meleagridis* and bacteria, to be used as media blanks. All the flasks were then sampled immediately after *H. meleagridis* inoculation (zero time point) and at 6, 18, 42, 66, 114, 142, and 166 h post inoculation (HPI). Sampling for each timepoint took roughly 15 min, with t0 corresponding to 0.25 HPI.

At each sampling period, including the media blanks, 4 mL of each T75 flask was filtered through Whatman^®^ Nuclepore™ Track-Etched Membranes with a 0.4 μm pore size (Sigma Millipore, Burlington MA USA). The filter was folded and placed in 2 mL cryovials and flash-frozen in liquid nitrogen. The cryovials were stored in −80 °C until the extraction process. Global metabolomics analysis was performed at the Biological and Small Molecule Mass Spectrometry Core (BSMMSC), University of Tennessee, Knoxville, TN, USA (RRID: SCR_021368). Using an acidic acetonitrile extraction procedure, metabolites were extracted from the filters using methanol, acetonitrile, and water (2:2:1) with 0.1% formic acid [14]. A Synergy Hydro-RP column (100 × 2 mm, 2.5 μm particle size) was used to separate metabolites based on retention time, with 10 μL of the sample injected into the Dionex UltiMate 3000 UPLC system (Thermo Fisher Scientific, Waltham, MA, USA). An Exactive™ Plus Orbitrap MS (Thermo Fisher Scientific, Waltham, MA, USA) was used for mass spectral analysis with negative-mode electrospray ionization, using an established method [15,16].

*Histomonas meleagridis* counts (histomonads/mL) were recorded at each timepoint for each flask using a hemocytometer. At each timepoint, 200 μL from each flask was collected, serially diluted ten-fold, and plated on Columbia blood agar with 5% sheep blood (Thermo Scientific™, USA) to detect the bacterial colony-forming units per ml of medium. The plates were incubated at 35 °C in a CO_2_ incubator, and the colonies were counted after 24 h incubation.

### 2.3. Data Analysis

The growth curves of undefined bacteria and *H. meleagridis* were generated in Microsoft Excel using the log of values (CFU/mL for bacteria or cells/mL for *H. meleagridis*). Mixed-model analysis and pairwise comparisons were performed using IBM SPSS statistics 27 to compare the different media used across various timepoints.

Following the preliminary ultra-high-performance liquid chromatography–high-resolution mass spectrometry (UHPLC-HRMS) global metabolomics method for water-soluble metabolites, raw spectral files were converted to mzML files using msConvert, a package from ProteoWizard [17,18]. These files were imported into an open-source software, Metabolomics Analysis and Visualization Engine (MAVEN2), to visualize extracted ion chromatograms (EICs) for data processing, in which peak areas were integrated for each identified metabolite [19,20]. Metabolites were identified in MAVEN by exact mass (±5 ppm mass accuracy) and chromatographic retention time from an in-house standard library. Peak areas were averaged for biological replicates. From these data, heatmaps and partial least squares discriminant analysis (PLS-DA) plots were generated. R (version 3.6.0) was used to generate heatmaps expressing log2 fold changes for each metabolite, and *p*-values were determined by a Student’s *t*-test. Prior to performing the PLS-DA analysis and generating volcano plots, the data were filtered using interquartile range (IQR), log-transformed, and Pareto-scaled using features in MetaboAnalyst 5.0 [21].

## 3. Results

### 3.1. Histomonas meleagridis Growth

**Figure 1:**
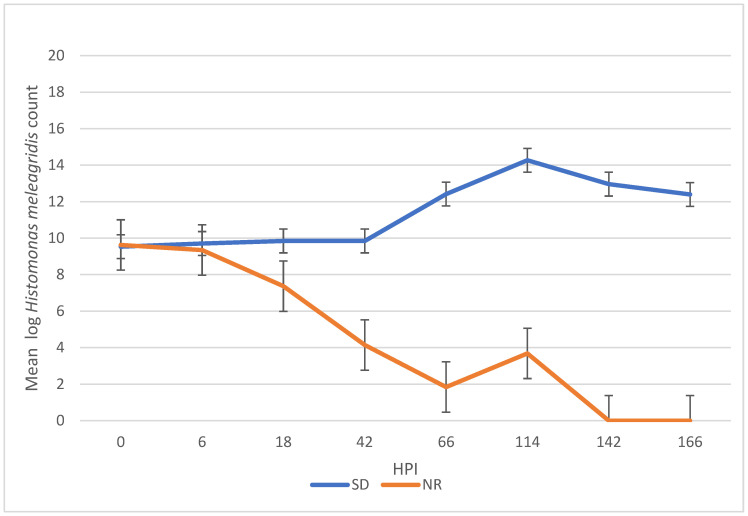
Growth curve of *Histomonas meleagridis* grown in Dwyer’s media with (SDM) and without (NR) rice starch. The mean of the log values is represented on the vertical axis and the hours post inoculation (HPI) on the horizontal axis.

There was a significant difference in *H. meleagridis* growth in SD compared to NR media. At 42, 66, 114, 142, and 166 HPI, the *H. meleagridis* mean log count was higher in the SD media than in the NR media (*p* < 0.001). A decline in *H. meleagridis* growth occurred approximately six hours after inoculation in the media lacking rice. In contrast, *H. meleagridis* grew considerably well in the SD media and reached the peak at 114 h, which was followed by a rapid decline (Figure 1).

### 3.2. Growth of Undefined Bacteria

**Figure 2:**
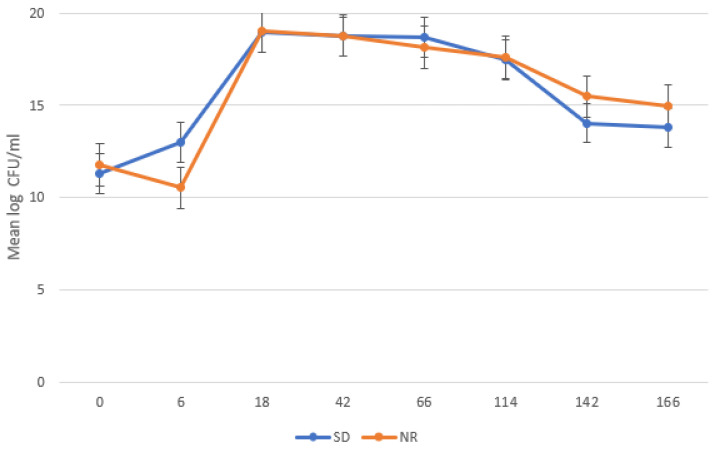
Growth curve of undefined bacteria in *Histomonas meleagridis* cultures using Dwyer’s media with (SDM) and without (NR) rice starch. The mean of the log values is represented on the vertical axis and the hours post inoculation (HPI) on the horizontal axis. “CFU” stands for colony-forming unit.

Bacterial growth showed a typical bacterial growth curve (Figure 2), with the lag phase ending at 6 HPI, the exponential phase occurring from 6 to 18 HPI, and a stationary phase occurring from 18 to 114 HPI. Bacteria declined starting at 142 HPI. There was no significant difference in mean log count of the bacteria in the SD and NR media, except at 6 HPI where the bacterial count was significantly higher in the SD media compared to the NR media (*p* = 0.002).

### 3.3. Metabolic Profile of H. meleagridis and Undefined Bacterial Populations in Dwyer’s Media with and Without Rice

From the intracellular metabolite analysis of the samples collected from the SD and NR media, there was a total of 170 metabolites identified (Figure S1). Heat mapping was used for visualization of the metabolite fold changes between the SD and NR media throughout the various timepoints. The greatest magnitude of change between the metabolite relative abundances is evident from 66 to 142 HPI, as indicated by the brightness of the fold changes in the heatmap, which is consistent with the greatest changes in magnitude of the HM growth curve. Almost all of the identified metabolites decreased in the NR media compared to the SD media at 66–142 HPI. There is a significant difference in the metabolites at 6 HPI between the SD and NR media, while there was a minimal difference in the blank media.

**Figure 3:**
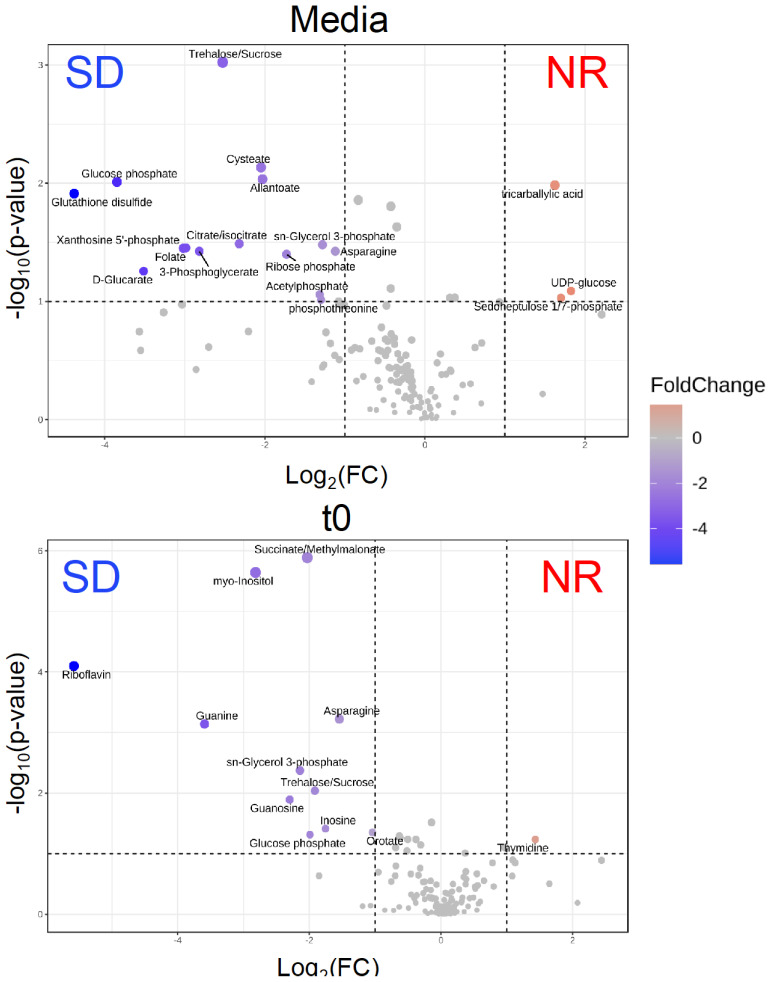
The volcano plots show that the intracellular metabolites are statistically and significantly different between *Histomonas meleagridis* and undefined bacteria in Dwyer’s media with (SD) and without (NR) rice starch. Fold change equals log_2_ (average relative abundance for NR/average relative abundance for SD). Red indicates that the metabolite has higher relative abundance in NR treatment, while blue indicates the metabolite has lower abundance in NR treatment. The metabolites were collected immediately at timepoint t0—representing ~0.25 h post infection—after the inoculation of *H. meleagridis*.

**Figure 4:**
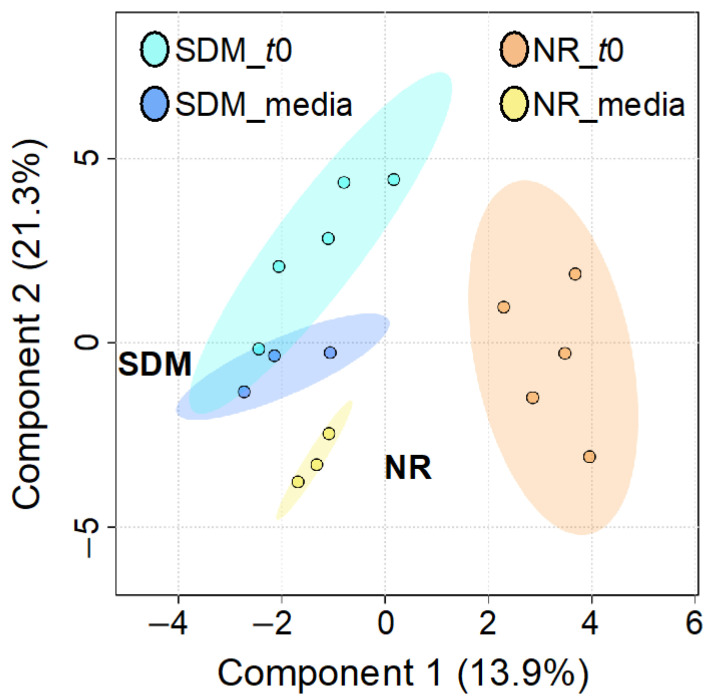
Partial least squares discriminant analysis (PLS-DA) of metabolites in Dwyer’s media with (SD) and without (NR) rice inoculated with *Histomonas meleagridis* and undefined bacterial population at blank and 0 HPI (t0). Ellipse represents 95% confidence interval.

**Figure 5:**
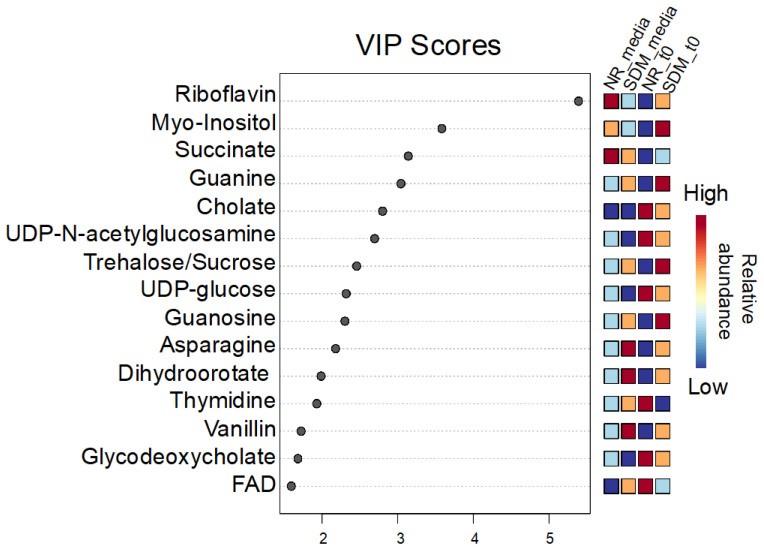
Variable importance in projection (VIP) scores for the top 15 metabolites contributing the most to the differences in the metabolic profile between Dwyer’s media with and without rice inoculated with *Histomonas meleagridis* and undefined bacteria in the media blank and 0 HPI. Metabolites with a VIP score over 1 drive the separation in the PLS-DA plot. Riboflavin has the highest VIP score in all 5 components (5.3907-1.1438).

**Figure 6:**
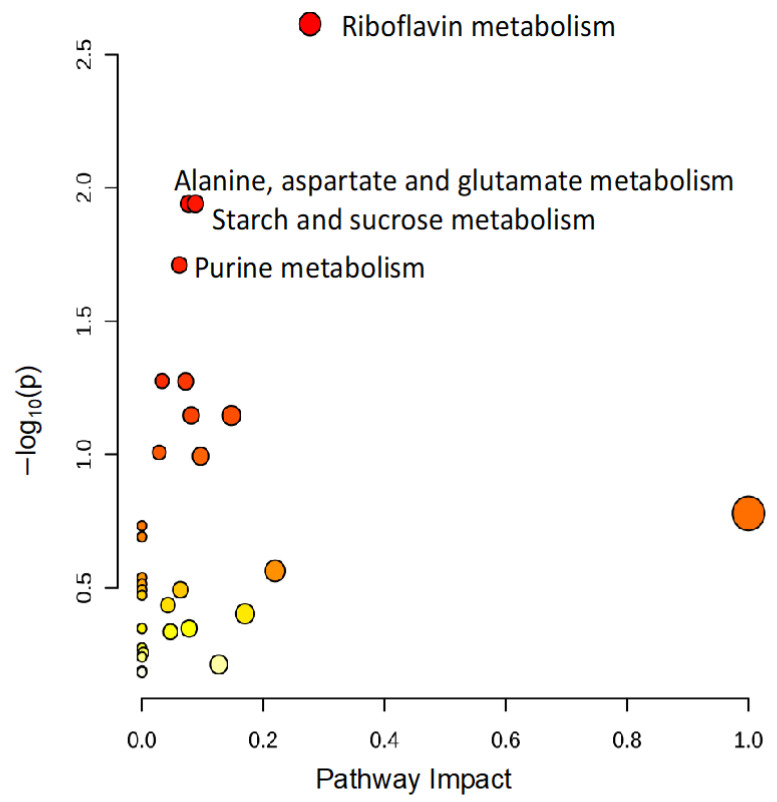
The pathway analysis reveals that riboflavin metabolism and pathways generating metabolic precursors of riboflavin are significantly altered based on media composition. All metabolites with variable importance in projection (VIP) scores >1 were used to conduct a pathway analysis to identify changes in metabolic pathways altered in NR media compared to SD media. Each circle represents a pathway, and the colors indicate the significance (*y*-axis), while the size depicts the pathway impact (*x*-axis). The more intense the shade of red, the lower the *p*-value, and the bigger the circle, the higher the impact of the pathway. Only pathways with *p* < 0.05 are labeled.

To identify metabolomic differences in microbes in the media before and immediately after (0.25 HPI) the inoculation of *H. meleagridis* and undefined bacteria, volcano plots (Figure 3) were generated. The differences in the media blanks between SD and NR are likely a reflection of the difference in media composition, and therefore available nutrients, provided to the microbes present in the media prior to the inoculation of *H. meleagridis*. This trend was further investigated using PLS-DA to identify differences in the metabolic profiles and the metabolites driving these differences (Figure 4). There were distinct metabolic profiles between the SD and NR media at blank and 0 HPI as indicated in Figure 4 by visual separation of groups. Riboflavin was the metabolite with the highest variable importance in the projection (VIP) score in the blank and 0 HPI (Figure 5). All the detected metabolites were assigned a VIP score, and any metabolite with a VIP score > 1 was deemed to significantly contribute to the separation of the groups (drives the differences in the metabolic profiles). There were 29 metabolites with VIP scores >1 (Figure S2), which were used for pathway analysis (Figure 6) to identify the metabolic pathways impacted most by media composition.

## 4. Discussion

Dwyer’s media has been used since 1970 for the cultivation of *H. meleagridis* and is the most routinely used media in laboratories today with minor modifications [7]. We used a modified formula of the original Dwyer’s media recipe to cultivate *H. meleagridis*. Our modified recipe contains rice starch and no chick embryo extract. We confirmed that rice starch is an essential component of Dwyer’s media. Omitting the rice starch or replacing it with other products greatly affected *H. meleagridis* growth [10]. Furthermore, the purity and rice granules’ size have some implications on *H. meleagridis* yield in media [11]. In our study, a metabolic analysis of the *H. meleagridis* intracellular metabolome grown in Dwyer’s media with and without rice was conducted to investigate specific nutrient requirements and, specifically, the role of rice starch in media. The cultures contained undefined bacteria that were inoculated in media when the *Histomonas* strain was originally isolated. Though it is not possible to separate bacterial metabolites from parasitic metabolites or determine the contribution from each population to the overall detected metabolite abundance, the heat map (S1) may suggest that *H. meleagridis* is responsible for driving the changes in the metabolic profile as the metabolite fold changes strongly correspond to the growth curve of *H. meleagridis*.

A mutualistic relationship between *Histomonas* and bacteria has been proposed but not a predator–prey relationship because *Histomonas,* like many other parasites, depends on the host to provide nutrients essential for survival [12]. Based on the current study, it is proposed that the bacteria may be playing a role by providing the *Histomonas* with riboflavin (B2), which is an essential vitamin required for many biological processes in the cell such as energy metabolism and fatty acid oxidation [22,23]. Higher abundance of a metabolite can either be attributed to increased production of the metabolite or decreased consumption. Bacteria are capable of producing riboflavin endogenously, but when there is an external source of riboflavin, they are equipped with the enzymes to transport riboflavin from exogenous sources [22]. M199 media that are used for making Dwyer’s media contains a very low amount of riboflavin at 0.00001 g/L.

Based on the data presented, we hypothesize that in the absence of rice starch supplementation, the bacteria are using de novo riboflavin biosynthesis, producing riboflavin from pentose phosphate and purine pathway metabolite precursors. This may explain why there is a higher abundance of riboflavin, sedoheptulose 1,7 phosphate, and IMP in the microbes in the NR media blanks. The media used in this experiment were prepared in clean non-sterile conditions, and although the bacteria levels in the media blank samples were lower than our limit of detection, the media left in the incubator for an additional day changed in color without the addition of any histomonads, suggesting the presence of living microbes. These microbes present in the media contributed to the differences observed in the SD and NR media blanks as these samples are representative of the intracellular metabolome of the initial microbial community. The increase in intracellular riboflavin in the NR media compared to the SD media may be attributed to the lack of a sufficient external source of riboflavin in NR for the bacteria, so the bacteria actively produced it. In the SD media, the white rice starch is a source of riboflavin providing an environment rich in riboflavin precursors, sedoheptulose 1,7 phosphate, and IMP, for the bacteria (Figures S3 and S4).

**Figure 7:**
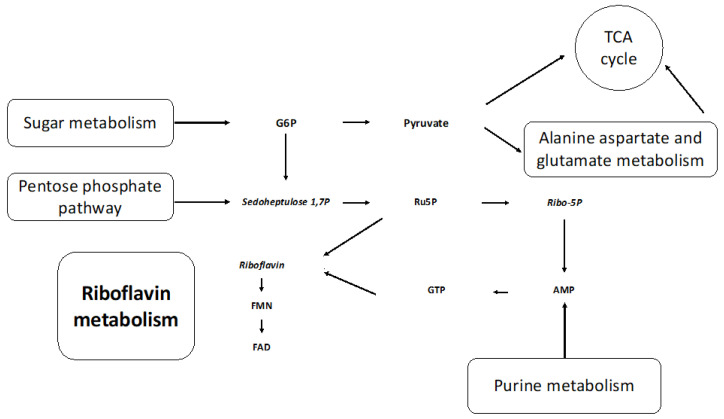
Riboflavin and metabolic precursors from interconnected metabolic pathways are altered by the omission of rice starch. G6P: glucose-6-phosphate, TCA: tricarboxylic citric acid, sedoheptulose 1,7P: sedoheptulose 1,7 phosphate, Ru5P: Ribulose 5-Phosphate, Ribo-5P: Ribose 5-phosphate, AMP: adenosine monophosphate, GTP: guanine triphosphate, FMN: flavin mononucleotide, FAD: flavin adenine dinucleotide. The metabolites in italics have increased in levels in the NR media compared to the SD media.

The metabolic pathways impacted most by media composition were riboflavin (riboflavin, FMN, and FAD), alanine, aspartate, glutamate (asparagine, 2-oxyglutarate, and succinate), starch, sucrose (UDP-glucose, sucrose, and trehalose), and purine (allantoate, dGMP, guanine, guanosine, and AMP) metabolism. The interconnection of these metabolic pathways, as shown in Figure 7, further supports our vitamin deficiency hypothesis (Figure S5). Metabolites involved in purine metabolism—guanosine and guanine—were lower in abundance in the NR culture at t0 (0.25 HPI). It has been established that purine salvage is essential for all obligate protozoan parasites, and rice starch is a known exogenous purine source [24]. It is likely that the mutualistic relationship between *H. meleagridis* and its bacterial host is mitigated when nutrients are limited (NR).

Furthermore, a recent review on the production of riboflavin in microorganisms reported the genes required to biosynthesize riboflavin: *Rib1*, *Rib2*, *Rib3*, *Rib4*, *Rib5*, and *Rib7* [25]. Using the NCBI BLAST search function, we searched the *H. meleagridis* genome for these genes [26]. However, there were no significant gene similarities found, suggesting that *H. meleagridis* is not capable of de novo riboflavin synthesis. Similarly, *Toxoplasma gondii*, an intracellular protozoan parasite, lacks the cellular machinery for de novo riboflavin biosynthesis [27]. *T. gondii* uses riboflavin salvage, depending on the host for this essential vitamin.

Riboflavin is a water-soluble vitamin, found in intestine of animals, that is necessary for flavin mononucleotide (FMN) and flavin adenine dinucleotide (FAD) biosynthesis [28]. Although riboflavin is present in the intestine and animal cells have specialized transporter proteins, animal cells are not capable of synthesizing riboflavin and depends completely on uptake of it from the intestine. Microbiota residing in the intestinal tract of animals are capable of synthetizing riboflavin. This happens either through a specific riboflavin biosynthetic pathway (RBP) or through using specialized importer proteins if the riboflavin is abundant in its microenvironment, thus saving energy [22,29].

Little is known about the growth requirement of *H. meleagridis*. However, bacterial secreted flavins are key metabolites in a variety of physiological processes in pro- and eukaryotes [22]. A deficiency in riboflavin would inhibit cellular growth, and metabolism in the forms of FMN and FAD syntheses would be negatively affected. This may explain why *H. meleagridis* is not able to grow in NR media, as FMN and FAD act as important cofactors and play a major role in energy production, cellular function, growth and development, neurotransmitter metabolism, and the metabolism of carbohydrates [22,30]. In fact, deficiency in vitamin B2 in birds presents as neurological symptoms [31]. Based on these data, it is likely that *H. meleagridis* requires riboflavin and other vitamins for its biological processes and depends on bacteria to provide this essential component as it is known that parasites in the gut depend on bacterial hosts for riboflavin.

*Histomonas meleagridis* infects the ceca and liver of birds [32], and the highest concentration of riboflavin is found in these two organs. More specifically, *H. meleagridis* causes lesions in bird ceca, and it has been found that the cecum of turkeys and chickens have double the riboflavin amount compared to the rest of the intestine [33]. However, fecal material from cecectomized birds contain approximately the same content of riboflavin as normal birds [33], which supports riboflavin’s role as an essential nutrient that, even in the absence of the ceca, the gut microbiota adjusts to provide the same amount of to the bird. Laying hens transfer riboflavin into the yolk and albumen and then to the embryos. The liver and eggs are an important source of vitamin B2, and chicken embryo extract has been used in *Histomonas* culture media as a replacement for hamster livers [7]. Diet may have an effect on the transmission dynamics of *H. meleagridis* in infected birds [34,35]; however, this effect is still poorly understood.

In conclusion, physiological changes are observed when rice starch is omitted from the media. The omission of rice starch inhibits *H. meleagridis* growth, yet it does not induce a change in the bacterial growth. Future research based on these data may allow for the propagation of axenic *H. meleagridis* cultures by manipulating and providing essential nutrients provided by the bacteria. Riboflavin is an important nutrient that may be supplemented by rice starch and metabolized by the bacteria into more biologically available products to be used by *H. meleagridis*. It is also an essential nutrient that is required for establishing axenic cultures of various organisms such as *Dictyostelium discoideum* [35], *Entamoeba histolytica* [36,37], and others [36,38,39]. Further studies on replacing rice starch with various forms and concentrations of riboflavin, its metabolic precursors, and other vitamins are warranted.

## Data Availability

The data presented in this study are available as Supplementary Materials (data_histomonas.csv).

## Supplementary Materials

The following supporting information can be downloaded at https://www.mdpi.com/article/10.3390/metabo14120650/s1, Figure S1: Heatmap of intracellular metabolites of *Histomonas meleagridis* and undefined bacteria in Dwyer’s media with (SD) and without (NR) rice starch showing the change in relative abundance of metabolites between the two media at various timepoints. Fold change equals log_2_ (average relative abundance for NR/average relative abundance for SD). Orange indicates that the metabolite has higher relative abundance in the NR treatment, blue indicates that the metabolite has lower abundance in the NR treatment, and black represents metabolites that do not change in relative abundance between the two treatments. The brightness represents the magnitude of change. *p*-values indicate if the change in relative metabolite abundance is significantly different between media conditions as follows: * *p* ≤ 0.1, ** *p* ≤ 0.05, *** *p* ≤ 0.01. NR: no rice media, SD: standard Dwyer’s media, AA: amino acid, and TCA: tricarboxylic acid cycle. Figure S2: All metabolites with a VIP score >1 from the PLS-DA analysis (Figure 4). Figure S3: Unnormalized average peak areas of riboflavin in Dwyer’s media with (SD) and without (NR) rice before (media) and after inoculation with *Histomonas meleagridis* and undefined bacteria. Vertical access represents the blank media (media) and the timepoints of sample collection from inoculated media. Figure S4: PLS-DA for t6. Figure S5: Volcano plot showing significantly altered metabolites. Fold change (NR/SD). Table S1: Data_histomonas.
