# Supplementary material for: Metabolic Profile of Histomonas meleagridis in Dwyer’s Media with and Without Rice Starch
Source: Metabolites. 2024 Nov 22;14(12):650. doi: 10.3390/metabo14120650 (PMC11676859; doi:10.3390/metabo14120650)
Supplement: Supplementary file 1 [file metabolites-14-00650-s001.zip › Supplementary.pdf]

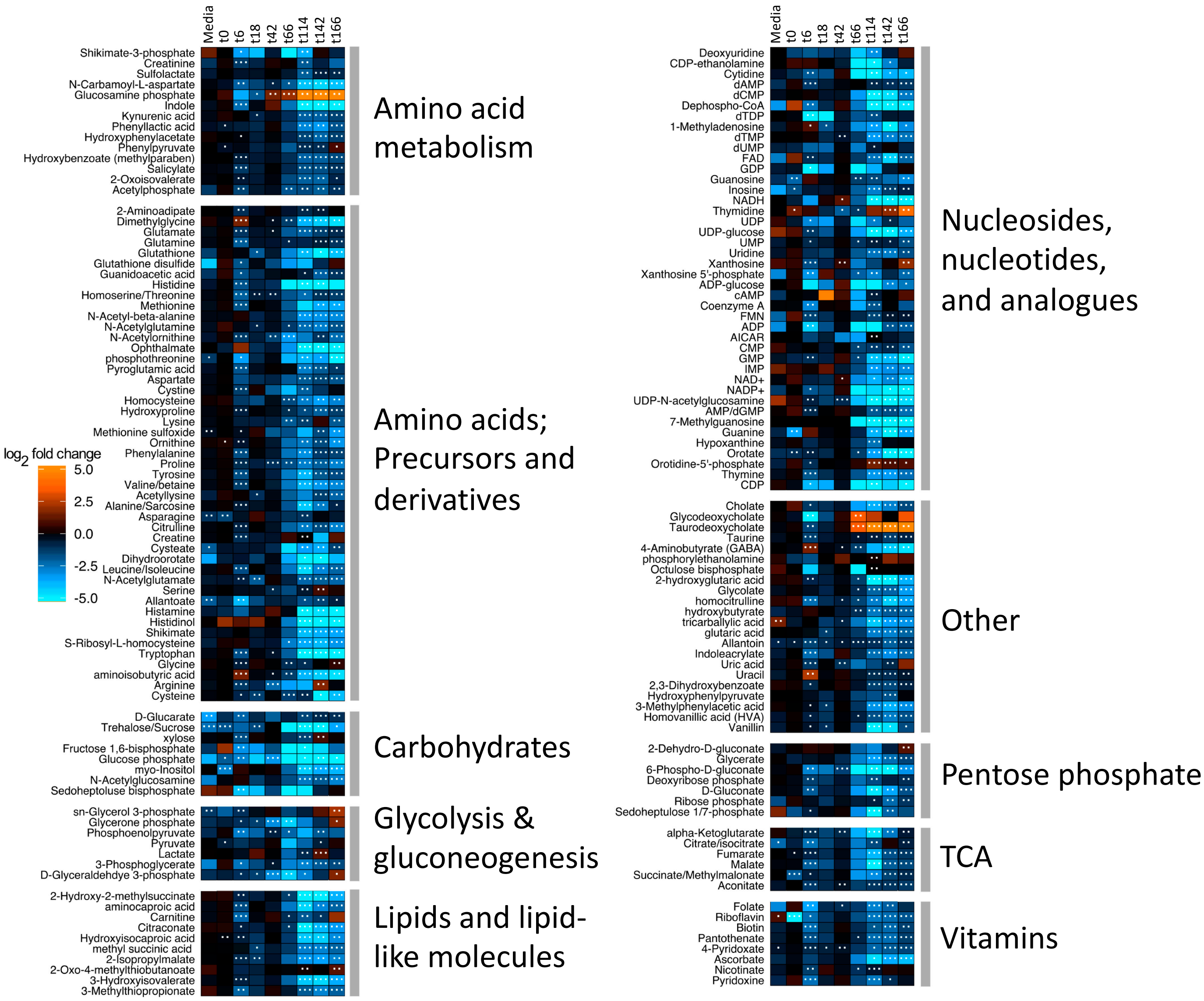

Figure S1: Heatmap of intracellular metabolites of Histomonas meleagridis and undefined bacteria in Dwyer's media with (SD) and without (NR) rice starch showing the change in relative abun-dance of metabolites between the two media at various timepoints. Fold change equals log2 (av-erage relative abundance for NR / average relative abundance for SD). Orange indicates metabo-lite has higher relative abundance in NR treatment, while blue indicates the metabolite has lower abundance in NR treatment, and black represents metabolite that do not change in relative abun-dance between the two treatments. The brightness represents the magnitude of change. P-values indicate if the change in relative metabolite abundance is significantly different between media conditions as follows, * ≤ 0.1, * * ≤ 0.05, * * * ≤ 0.01. NR is no rice media, SD is standard Dwyer's media, AA is amino acids and TCA is tricarboxylic acid cycle.

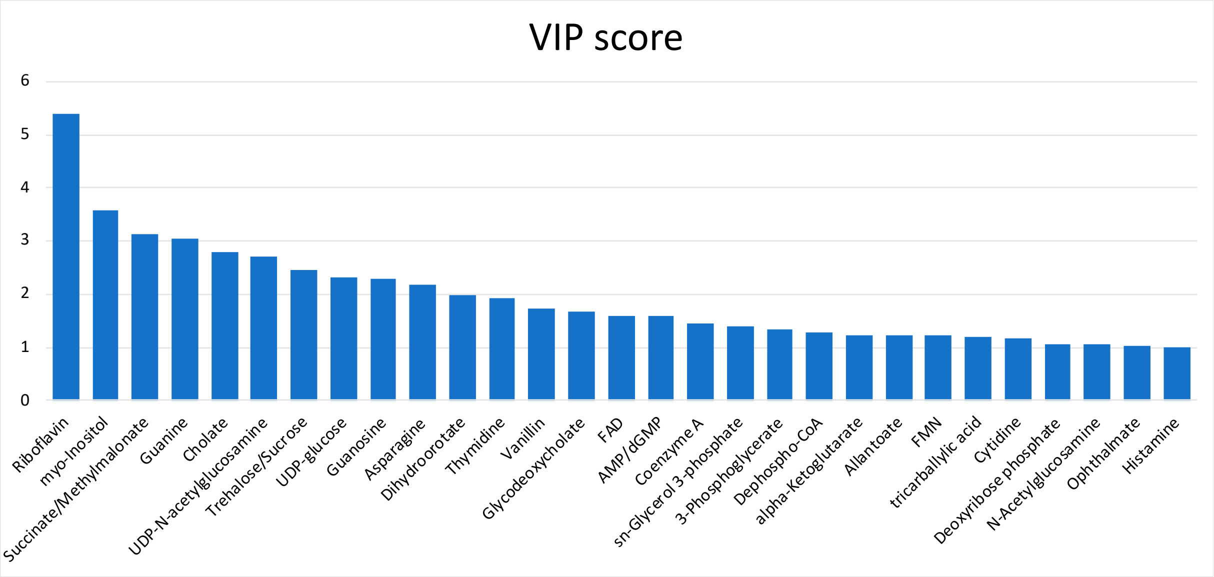

S2: All metabolites (29) with a variable importance in projection score (VIP) score >1 from PLS-DA analysis (Figure 4).

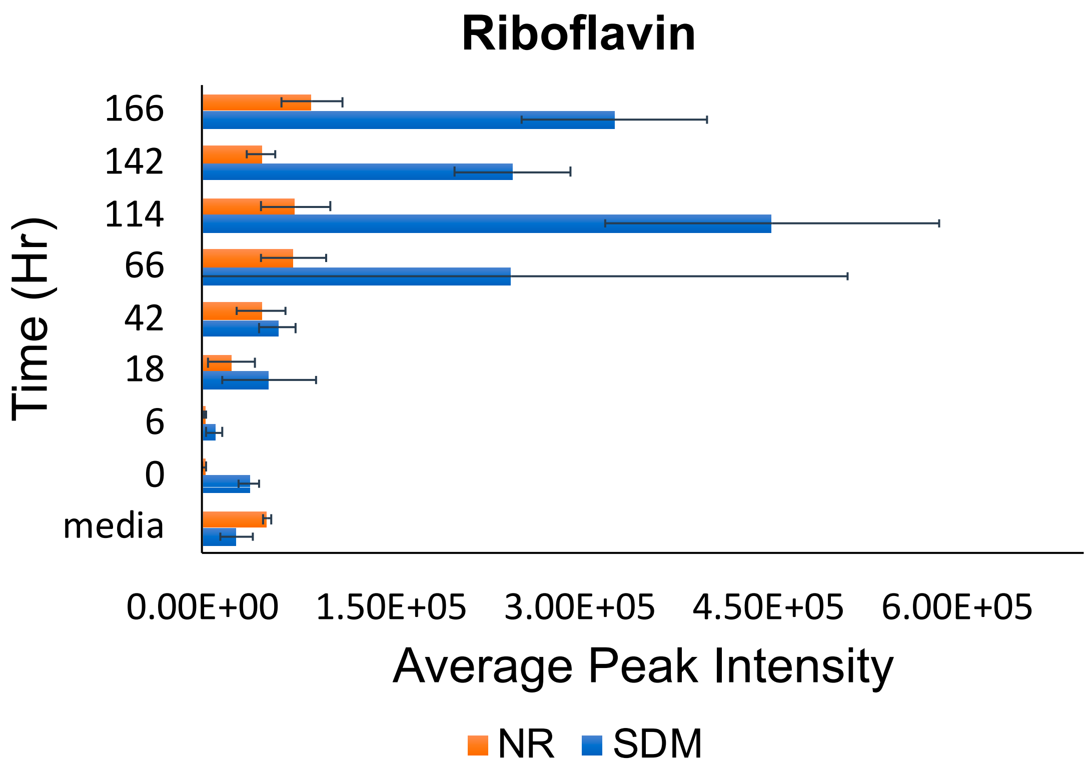

S3: Unnormalized average peak areas of riboflavin in Dwyer's media with (SD) and without (NR) rice before (media) and after inoculation with *Histomonas meleagridis* and undefined bacteria. Vertical access represents the blank media (media) and the timepoints of sample collection from inoculated media.

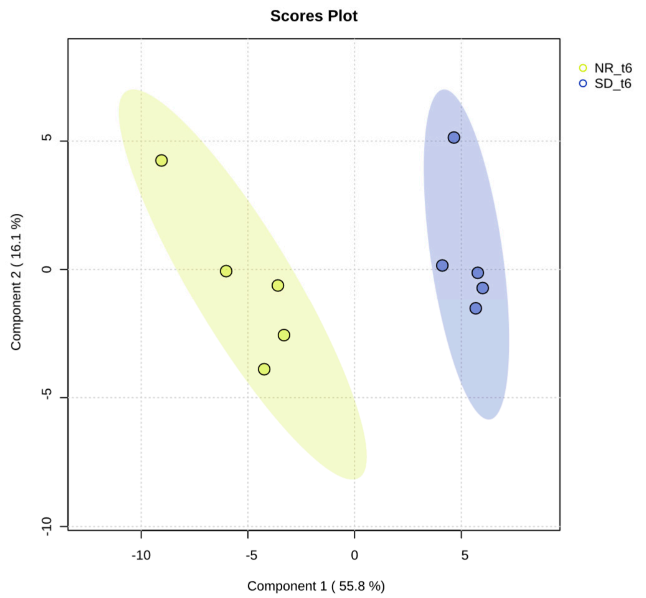

S4: PLSDA for t6

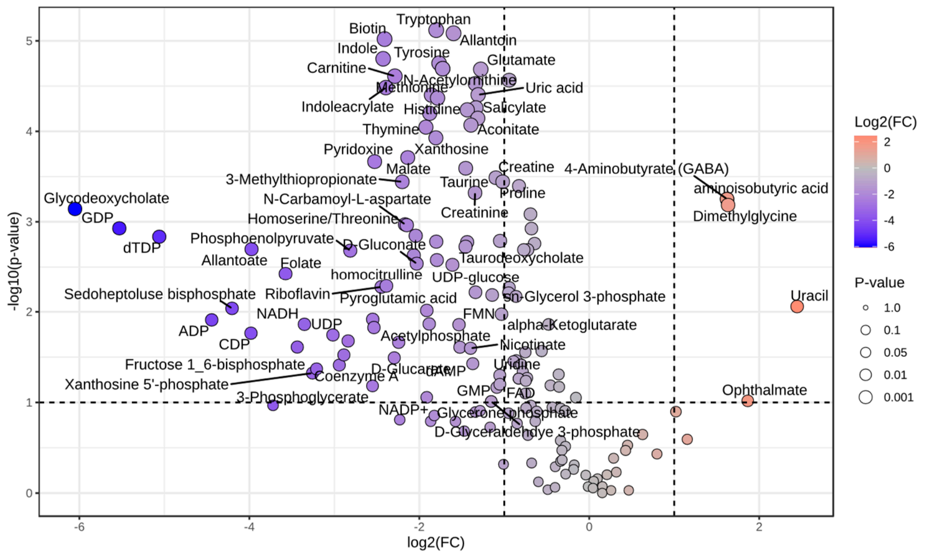

S5: Volcano plot showing significantly altered metabolites. Foldchange (NR/SD).
